# Supplementary material for: Evaluation of the use of GeneXpert MTB/RIF in a zone with high burden of tuberculosis in Thailand
Source: PLoS One. 2022 Jul 27;17(7):e0271130. doi: 10.1371/journal.pone.0271130 (PMC9328536; doi:10.1371/journal.pone.0271130)
Supplement: S1 File — (PDF) [file pone.0271130.s001.pdf]

QUESTIONNAIRES (ENGLISH) Part I

Code No………………..

This questionnaires composes with 4 sections which use to collect data in among TB patients with smear negative and high risk of MDR-TB who had been screened by the GeneXpert MTB/RIF in during time September 2015 to February 2018 in TB clinic of 5 areas in this study

Section I, II. Socio-demographic and back ground information

Explanation: Fill the answer in the right box

1. Age (Completed years) ☐
2. Sex ☐
   (i) Male
   (ii) Female
3. Address ☐
   (i) Urban
   (ii) Rural
   (iii) Others city (Please specify)…………………………
4. Ethnicity ☐
   (i) Thai
   (ii) Laos
   (iii) Cambodia
   (iv) Chinese
   (v) Others (Please specify)…………………………….
5. Marital Status ☐
   (i) Single
   (ii) Married
   (iii) Divorced
   (iv) Widowed
   (v) Separated
   (vi) Others (Please specify)……………………………..

6. Religions ☐

(i) Buddha

(ii) Christian

(iii) Muslim

(iv) No religions

(v) Other

7. Occupation ☐

(i) Agriculture

(ii) Laborers

(iii) Owned business/ self-employee

(iv) Government staff

(v) Unemployed

8. Education ☐

(i) Illiterate

(ii) Can read and write

(iii) Primary school level

(iv) Middle school level

(v) High School level

(vi) Graduate and above

9. Number of family members ☐

10. Type of family ☐

(i) Nuclear

(ii) Extended

(iii) Stay with friends

(iv) Other (Please specify)……………………………..

11. DOT personal ☐

(i) Health care workers

(ii) Public health volunteer

(iii) Family member

(iv) Other (Please specify)……………………………..

12. Underlying diseases ☐
   (i) Old TB
   (ii) DM
   (iii) Hypertension
   (iv) CRF
   (v) Other (Please specify)…………………………………

13. MDR-TB household contact/ MDR-TB patients contact history? ☐
   (i) Yes
   (ii) No

14. TB household contact/ TB patients contact history? ☐
   (i) Yes
   (ii) No

15. HIV infection ☐
   (i) Yes
   (ii) No

Section III; Laboratory process and details for diagnosis;

16. Typed of registered (Pul TB) ☐
   (i) MDR-TB
   (ii) New smear negative TB
   (iii) Relapsed
   (iv) Defaulted
   (v) Failed
   (vi) Others…………………………….

17. Signs & symptoms ☐
   (i) In TB criteria
   (ii) Non TB criteria

18. X-rays result ☐

(i) Normal

(ii) Ab infiltration

(iii) Ab cavity

(iv) Ab others……………………………….

19. AFB result ☐

(i) AFB +

(ii) AFB -

20. Type of laboratory screening used (Physician's order) (> 1 answer)

(i) AFB ☐

(ii) AFB and GeneXpert MTB/RIF ☐

(iii) AFB and TB culture/LPA ☐

(iv) Others...................................... ☐

21. GeneXpert MTB/RFP results

(i) M Detected with RR non Detected TAT =….Hr. or …..days ☐

(0) Dx Non TB

(1) Dx TB with FLD

(2) Dx TB without FLD

(ii) M non Detected ☐

(0) Dx Non TB

(3) Dx TB with FLD

(4) Dx TB without FLD

(iii) M Detected with RR Detected TAT =….Hr. or …….. ☐

(0) non Dx MDR-TB

(1) Dx MDRTB with SLD

(2) Dx MDRTB without SLD

(3) Dx RR-TB with SLD

(4) Dx RR-TB without SLD

(5) Others (Please specify)

22. TB culture results TAT =…………….Hr. or …………..days ☐ ☐

(i) Growth

(1) Dx TB without FLD

(2) Dx MDRTB with SLD

(ii) No growth

23. DST results TAT =…………….Hr. or …………..days ☐

(i) Mono Resistance

(ii) I,R Resistance

(iii) I, R, other Resistance

Section 4; Treatment history/ Physician recorded;

24. Period I; TAT time measures from once sputum specimen had been tested by the Xpert assays until got the result =………..…..hr. or…..………days

25. Period II. a; time when patients had started initial empirical MDR-TB treatment after the physician received the GeneXpert MTB/RFP result. =…..hr. or….days

26. Period II.b; time when started initial MDR-TB treatment after got the conventional culture and DST results had been confirmed. =…………….hr. or…………days

Types of lab confirm; (i) LPA, (ii) Culture, (iii) DST, (iv) Others ☐

27. Period III; measures from time when sputum specimen had been tested by

(i) LPA,

(ii) Culture/DST until got the result. =…………….hr. or…………days ☐

28. TB Diagnosis by ☐

(i) CXR result and GeneXpert result Mdetected

(ii) CXR result and without GeneXpert result (M not detected)

(iii) CXR result with lab conventional methods confirm

(iv) CXR result with others lab confirmed

29. MDR-TB Diagnosis by (> 1 answer)

(i) CXR result with GeneXpert M non detected ☐

(ii) CXR result with GeneXpert M detected and ☐

(1) RR detected (0) RR non detected ☐

(iii) CXR result with TB culture confirmed ☐

(iv) CXR result with TB culture and DST confirmed ☐

(v) CXR result with LPA confirmed ☐

(vi) CXR result with others lab confirmed…………………… ☐

QUESTIONNAIRES FOR IN-DEPTH INTERVIEW (ENGLISH) Part II.

Code No.....................

Section I; Socio-demographic and back ground information

1. Hospital areas ☐
   - (i) Khon Kaen hospital
   - (ii) Kalasin hospital
   - (iii) Roi-et hospital
   - (iv) Surin hospital
   - (v) Sisaket hospital
2. Age (Completed years) ☐
3. Sex ☐
   - (i) Male
   - (ii) Female
4. Occupation ☐
   - (i) Physician
   - (ii) Nurse
   - (iii) Others (Please specify)………………………..
5. Position at work ☐
   - (i) Professional
   - (ii) Technician
   - (iii) Others (Please specify)………………………..
6. Marital Status ☐
   - (i) Single
   - (ii) Married
   - (iii) Divorced
   - (iv) Widowed
   - (v) Separated
   - (vi) Others (Please specify)…………………………

7. Religions ☐

(i) Buddha

(ii) Christian

(iii) Muslim

(iv) No religions

(v) Other

(i) Certificate

(ii) Bachelor degree

(ii) Master degree

(iii) Doctorates

9. Working experience in TBC ☐

(i) < 1 year

(ii) 1- 5 Years

(iii) 6-10 Years

(iv) > 10 Years

In-depth interview questionnaires

1. What do you think about GeneXpert?

2. In term of the diagnosis for TB, MDR-TB, in your opinion, is the GeneXpert has beneficent? How?

3. Will you use the GeneXpert to be the main of TB, MDR-TB diagnosis?

4. How much you trust for the Xpert result compare with the conventional method results?

5. Is it possible that the GeneXpert will be replaced the conventional methods for TB,MDR-TB diagnosis further? Why?

6. Do you have any comments about the GeneXpert MTB/RIF?

7. In your opinion, do you think if the GeneXpert appropriates to be at the point of care? Why?
