# Supplementary material for: Evaluation of the use of GeneXpert MTB/RIF in a zone with high burden of tuberculosis in Thailand
Source: PLoS One. 2022 Jul 27;17(7):e0271130. doi: 10.1371/journal.pone.0271130 (PMC9328536; doi:10.1371/journal.pone.0271130)
Supplement: Attachment — Submitted filename: PONE-D-22-0-1562 Comments.docx [file pone.0271130.s002.docx]

Manuscript title: Evaluation of the use of GeneXpert MTB/RIF in a zone with high burden of tuberculosis in Thailand

Date: 1 March, 2022

The authors described the application of GeneXpert MTB/RIF assay in tuberculosis endemic area in Thailand. The manuscript is well written. Please consider the following comments for further improvement of the manuscript.

1. Please provide details of data collection procedures including collection of secondary data, interview of physicians and nurses.
2. In ethical considerations, also mention written consent procedure from the participants before data collection.
3. How did you define suspected TB cases? Among suspected cases, 21.8% had no signs and symptoms related to TB. How were they suspected of TB since they had no signs and symptoms?
4. Please summarized the opinions of clinicians on advantages, disadvantages and limitations of GeneXpert MTB/RIF to few sentences, and keep only one or two verbatim.
