# Supplementary material for: Evaluation of the use of GeneXpert MTB/RIF in a zone with high burden of tuberculosis in Thailand
Source: PLoS One. 2022 Jul 27;17(7):e0271130. doi: 10.1371/journal.pone.0271130 (PMC9328536; doi:10.1371/journal.pone.0271130)
Supplement: Attachment — Submitted filename: Comments.docx [file pone.0271130.s003.docx]

Reviewer’s comments

1. Recommendation: Manuscript can be accepted with Minor Revisions
2. Manuscript is technically sound
3. Statistical analysis: I don't know
4. Does the manuscript adhere to the PLOS Data Policy: Yes
5. Is the manuscript presented in an intelligible fashion and written in standard English: Yes
6. Would you like your identity revealed to the authors of this submission: Yes
7. Do you have any potentially competing interests: No
