# Supplementary material for: Evaluation of the use of GeneXpert MTB/RIF in a zone with high burden of tuberculosis in Thailand
Source: PLoS One. 2022 Jul 27;17(7):e0271130. doi: 10.1371/journal.pone.0271130 (PMC9328536; doi:10.1371/journal.pone.0271130)
Supplement: Attachment — Submitted filename: Response to Editors and Reviewers.docx [file pone.0271130.s004.docx]

***Responses to the Editor and the Reviewers***

***Journal Requirements:***

1. Please ensure that your manuscript meets PLOS ONE's style requirements, including those for file naming.

Response: Our manuscript has been rechecked before resubmission, following PLOS ONE’s style requirements.

2. Please provide additional details regarding participant consent. In the ethics statement in the Methods and online submission information, please ensure that you have specified (1) whether consent was informed and (2) what type you obtained (for instance, written or verbal, and if verbal, how it was documented and witnessed). If your study included minors, state whether you obtained consent from parents or guardians. If the need for consent was waived by the ethics committee, please include this information. If you are reporting a retrospective study of medical records or archived samples, please ensure that you have discussed whether all data were fully anonymized before you accessed them and/or whether the IRB or ethics committee waived the requirement for informed consent. If patients provided informed written consent to have data from their medical records used in research, please include this information.

Response: Thank you for your suggestions. Reviewer#1 had a similar suggestion. We have added a new sub-heading on “data collection procedures” in the Materials and Methods section. Under this additional sub-heading, we describe how we collected the data and acquired informed consent in for both quantitative and qualitative parts of the study. (Lines 158-183)

3. Please note that in order to use the direct billing option the corresponding author must be affiliated with the chosen institute. Please either amend your manuscript to change the affiliation or corresponding author, or email us at plosone@plos.org with a request to remove this option.

Response: All billing should be sent to the corresponding author, Dr. Jaranit Kaewkungwal, who is affiliated with Mahidol University.

4. We note that Figure S1 in your submission contain [map/satellite] images which may be copyrighted. All PLOS content is published under the Creative Commons Attribution License (CC BY 4.0), which means that the manuscript, images, and Supporting Information files will be freely available online, and any third party is permitted to access, download, copy, distribute, and use these materials in any way, even commercially, with proper attribution. We require you to either (1) present written permission from the copyright holder to publish these figures specifically under the CC BY 4.0 license, or (2) remove the figures from your submission.

Response: Thank you for your comment. We have removed Figure 1 from the manuscript. (Lines 119-120, 126-127)

5. Please review your reference list to ensure that it is complete and correct. If you have cited papers that have been retracted, please include the rationale for doing so in the manuscript text, or remove these references and replace them with relevant current references

Response: We have rechecked our references, following PLOS ONE guidelines.

***Reviewers' comments:***

***Reviewer #1:***

1. Please provide details of data collection procedures including collection of secondary data, interview of physicians and nurses.

Response: Thank you for your suggestion. We have added “Data Collection Procedures” to the Materials and Methods section, elaborating how we obtained data for both parts of the study. (lines 158-183)

2. In ethical considerations, also mention written consent procedure from the participants before data collection.

Response: Thank you. We have added consent procedures to the data collection section for both parts of the study. (lines 158-183)

3. How did you define suspected TB cases? Among suspected cases, 21.8% had no signs and symptoms related to TB. How were they suspected of TB since they had no signs and symptoms?

Response: The definition of “suspected TB cases” has been inserted into the Materials and Methods section. We have also renamed the sub-heading “Study design” to become “Study design and case definition”. (lines 95-114)

4. Please summarized the opinions of clinicians on advantages, disadvantages and limitations of GeneXpert MTB/RIF to few sentences, and keep only one or two verbatim.

Response: Thank you. We have revised the section “Opinions regarding the use of GeneXpert MTB/RIF for PTB and MDR-TB diagnosis”. We have summarized the opinions of clinicians on advantages, disadvantages and limitations to a few sentences, and kept a few verbatim, as suggested. (lines 383-467)

***Reviewer #2:***

The manuscript is well written. Results are presented in little confusing manner and are need to reframed in more appropriate pattern. References can be updated with more recent research studies. Overall manuscript is satisfactory.

Response:: Thank you for your suggestions. We have revised parts of the Results section, particularly the part about “Opinions” of clinicians, as also suggested by Reviewer#1. To clarify the frequently used terms in TB case management, prevention, and control measures, we have added an “Abbreviations” section at the end of the manuscript (Lines 633-641), and we reduced the less frequently used abbreviations. We also added 2 recent references regarding GeneXpert to the Discussion section. (lines 498-500, 559-563)
